# Exploring the Therapeutic Potential and Toxicological Risks of Four Ethnomedicinal Plants from Hakkâri (Southeastern Turkey): A First Comprehensive Analytical and Microstructural Evaluation

**DOI:** 10.3390/plants14213243

**Published:** 2025-10-22

**Authors:** Gül Görmez

**Affiliations:** Faculty of Health Sciences, Nutrition and Dietetics Department, Van Yuzuncu Yil University, 65100 Van, Turkey; gulgormez@yyu.edu.tr

**Keywords:** medicinal plants, *Daphne mucronata*, *Ferula communis*, *Heracleum persicum*, Tragopogon coloratus, health risk assessment, cancer risk, heavy metal contamination, phenolic acids, rutin

## Abstract

Medicinal plants have long been used for therapeutic purposes in the mountainous Hakkâri region of southeastern Türkiye. This study presents an integrated toxicological risk and therapeutic assessment of four ethnomedicinal species—*Daphne mucronata* Royle, *Ferula communis* L., *Heracleum persicum* Desf., and *Tragopogon coloratus* C.A.Mey—based on their flavonoid and phenolic composition, elemental content, and antioxidant capacity. To the best of our knowledge, this is the first study to integrate multiple analytical platforms—including HPLC, ICP-OES, AAS, UV-Vis spectrophotometry, and SEM/EDX—to assess both the therapeutic potential and toxicological risks of these ethnomedicinal species. Although a complete phytochemical profile was not the objective of this study, selected phenolic compounds and antioxidant capacity were evaluated to highlight bioactivity, while heavy metal-based risk assessment was prioritized given public health relevance. Antioxidant capacity was measured using DPPH, ABTS, and CUPRAC assays, while human health risks were quantified through Estimated Daily Consumption (EDC), Target Hazard Quotient (THQ), Hazard Index (HI), and Carcinogenic Risk (CR). The results revealed a dual nature: *Heracleum persicum* exhibited the strongest antioxidant activity, correlating with its high phenolic content, while *Daphne mucronata* showed elevated toxic metals exceeding WHO/FAO thresholds. Overall, the findings emphasize the importance of combining ethnobotanical knowledge with robust analytical tools for safe medicinal plant usage.

## 1. Introduction

The Hakkâri province, located in southeastern Türkiye, may represent one of the most diverse ethnobotanical regions of Anatolia, hosting a wide range of edible and medicinal plant species that local communities have used for generations. Traditional healing practices in this region address a variety of health problems, such as toothaches, gastrointestinal disturbances, rheumatism, wounds, and burns. Many of these plants are also integrated into regional cuisine, for instance as seasonal herbs or flavoring ingredients in local cheeses. Despite their long-standing cultural and therapeutic importance, there has been a notable lack of systematic research examining their biochemical composition and potential toxicological risks. This absence is critical, as plants can naturally absorb and retain both essential and toxic elements from the soil and atmosphere. Consequently, investigating their phytochemical composition, antioxidant potential, and elemental accumulation is necessary to evaluate both their health-promoting and risk-bearing properties.

This research focuses on four ethnomedicinal plants commonly used in the Hakkâri region: *Daphne mucronata* Royle (Thymelaeaceae), *Ferula communis* L. (Apiaceae), *Heracleum persicum* Desf. (Apiaceae), and *Tragopogon coloratus* C.A.Mey. (Asteraceae). These species were selected based on their frequent use in traditional remedies and dietary practices. *Daphne mucronata* has been associated with anticarcinogenic, antioxidant, and antibacterial activities [1,2,3]. Ethnobotanical records indicate its application for muscular pain, eye and skin disorders, and intestinal parasites [4]. Local interviews confirmed its use for dental pain and mouth sores, generally as an infusion. *Ferula communis* has shown antioxidant [5] and antibacterial [6] activity and is traditionally utilized for hormonal regulation and menopausal discomfort [7]; in local cuisine, it is also incorporated into herbed cheeses and burn ointments. *Heracleum persicum*, rich in flavonoids, furanocoumarins, and other phenolic compounds, displays antidiabetic, anti-inflammatory, and cytotoxic activities [8,9,10]; it is used regionally for snakebite treatment and digestive support. Plants of the Tragopogon genus are known for antioxidant, antibacterial, hepatoprotective, and wound-healing effects [11,12,13,14,15,16], and *Tragopogon coloratus* is traditionally employed in Hakkâri for stomach pain and ulcers [17].

The accumulation of heavy metals such as cadmium (Cd), lead (Pb), chromium (Cr), copper (Cu), and arsenic (As) in medicinal plants represents a potential health concern. These elements can enter plant tissues through root uptake or atmospheric deposition and may reach hazardous levels, depending on environmental conditions. In the semi-arid soils of Southeastern Anatolia, trace elements are geochemically present at low yet biologically active concentrations [18,19,20]. As a result, plants consumed as food or medicine can become a source of heavy metal exposure. The chronic intake of contaminated plant materials has been associated with enzyme inhibition [21], oxidative stress via reactive oxygen species (ROS) formation [22], and neurological, renal, and carcinogenic effects [23,24]. Several studies from Türkiye and other countries have reported that some herbs and spices contain Cd, Pb, Ni, Cr, and Cu levels exceeding international safety limits [22,25,26,27], emphasizing the need for strict evaluation of both their phytochemical benefits and toxicological risks [28,29].

To the best of our knowledge, this study provides the first comprehensive evaluations of the selected phenolic acids, elemental compositions, antioxidant capacities, and health risk parameters of *Tragopogon coloratus*, *Heracleum persicum*, *Ferula communis*, and *Daphne mucronata* collected from the Hakkâri region. Considering their dual role as medicinal and dietary resources, understanding the balance between their therapeutic value and toxic potential is crucial. The present work aims to support the safe and sustainable use of these culturally significant species, ensuring the continuation of traditional practices while minimizing potential health hazards.

## 2. Materials and Methods

### 2.1. Study Area

**Figure 1:**
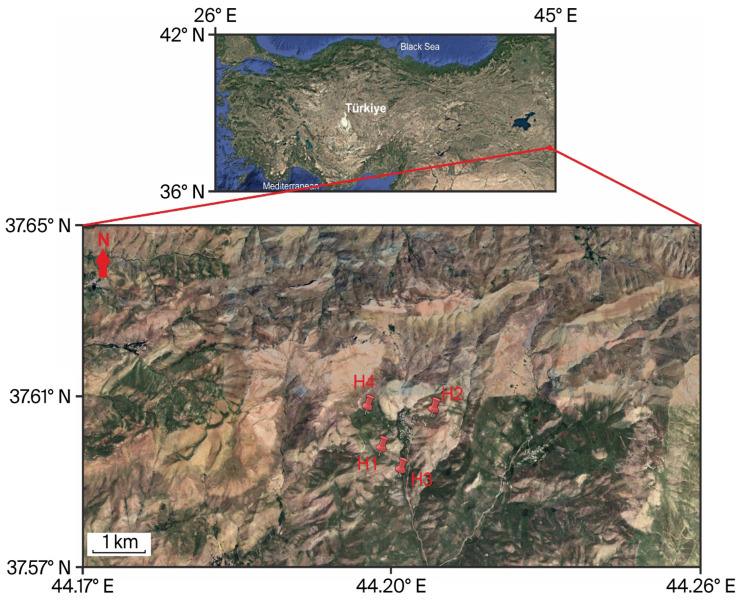
The location of the research area. Sampling sites of the investigated species are indicated as follows: H1; *Daphne mucronata*, H2; *Ferula communis*, H3; *Heracleum persicum*, H4; *Tragopogon coloratus*.

The plants used in this research were collected in Hakkâri Province, southeastern Anatolia, Türkiye, (37.65° N, 44.26° E), during May and June 2024 (Figure 1). Hakkâri, a rugged mountainous province covering approximately 7121 km^2^, had a population of 278,218 in 2022 according to the Turkish Statistical Institute (TÜİK) [30]. The province experiences a continental climate characterized by prolonged, frigid winters and brief, arid summers. According to the Turkish State Meteorological Service (MGM), the mean annual temperature is 10.4 °C, and the average yearly precipitation is 793 mm, with spring and autumn being the wettest seasons [31]. Due to the semi-arid climate and terrain, dust and flying particles are likely to move through and settle on plants. This may help heavy metals build up on their surfaces. According to traffic data from the General Directorate of Highways (KGM) for 2024, Hakkâri still has a low daily vehicle density. However, some road segments accommodate up to 4700 vehicles per day, with significant heavy-duty traffic [32]. All plant specimens were taxonomically verified by expert botanists. *Daphne mucronata*, *Ferula communis*, and *Heracleum persicum* were identified and confirmed by Prof. Dr. Nasip Demirkuş, whereas *Tragopogon coloratus* was confirmed by Prof. Dr. Murat Ünal. Voucher specimens were deposited in the Herbarium of Van Yüzüncü Yıl University (VANF) for future reference.

The plant gathering took place from May to July 2024, coinciding with the natural vegetation period.

### 2.2. Chemicals and Reagents

Methanol (CH_3_OH, HPLC grade), ethanol (C_2_H_6_O, HPLC grade), potassium persulfate (K_2_S_2_O_8_, ≥99% purity), copper(II) chloride dihydrate (CuCl_2_·2H_2_O), and nitric acid (HNO_3_, 65%, ultrapure, ICP grade) were purchased from Merck KGaA (Darmstadt, Germany). Ultrapure water (Milli-Q purification system, Merck Millipore, Burlington, MA, USA; resistivity ≥18.2 MΩ·cm) was used in all HPLC analyses and solution preparations. Before use, all solutions were filtered through 0.45 µm membrane filters (Sartorius AG, Göttingen, Germany) to eliminate particulates, and chemicals were stored under appropriate conditions to protect them from light, air, and moisture. Antioxidant standards and radical reagents, including 2,2-diphenyl-1-picrylhydrazyl (DPPH), 2,2′-azino-bis(3-ethylbenzothiazoline-6-sulphonic acid) (ABTS) diammonium salt, Trolox (6-hydroxy-2,5,7,8-tetramethylchroman-2-carboxylic acid), α-tocopherol (vitamin E), butylated hydroxytoluene (BHT), phosphate buffer (0.1 M, pH 7.4), ammonium acetate buffer (1.0 M, pH 7.0), and neocuproin (2,9-dimethyl-1,10-phenanthroline), were obtained from Sigma-Aldrich Chemical Co. (St. Louis, MO, USA). Phenolic and flavonoid reference standards, including gallic acid (3,4,5-trihydroxybenzoic acid), protocatechuic acid (3,4-dihydroxybenzoic acid), vanillic acid (4-hydroxy-3-methoxybenzoic acid), chlorogenic acid (3-O-caffeoylquinic acid), p-coumaric acid (4-hydroxycinnamic acid), o-coumaric acid (2-hydroxycinnamic acid), ferulic acid (4-hydroxy-3-methoxycinnamic acid), and rutin (quercetin-3-O-rutinoside) were obtained from Sigma-Aldrich Chemical Co. (St. Louis, MO, USA) (purity 95–99%, HPLC grade). According to the supplier specifications, the purity of these standards ranged between 94% and 99% (HPLC or analytical grade). All reference compounds were freshly prepared in methanol before HPLC injection and stored at 4 °C in amber vials to prevent degradation. Acetonitrile (98%, analytical grade) was obtained from Fluka Analytical, Honeywell Research Chemicals (Buchs, Switzerland). Before ICP-OES and AAS measurements, technique validation was carried out using a certified reference material (NIST SRM 1570a spinach leaves; National Institute of Standards and Technology, Gaithersburg, MD, USA) to ensure the accuracy and reliability of elemental determinations.

### 2.3. Plant Material

**Figure 2:**
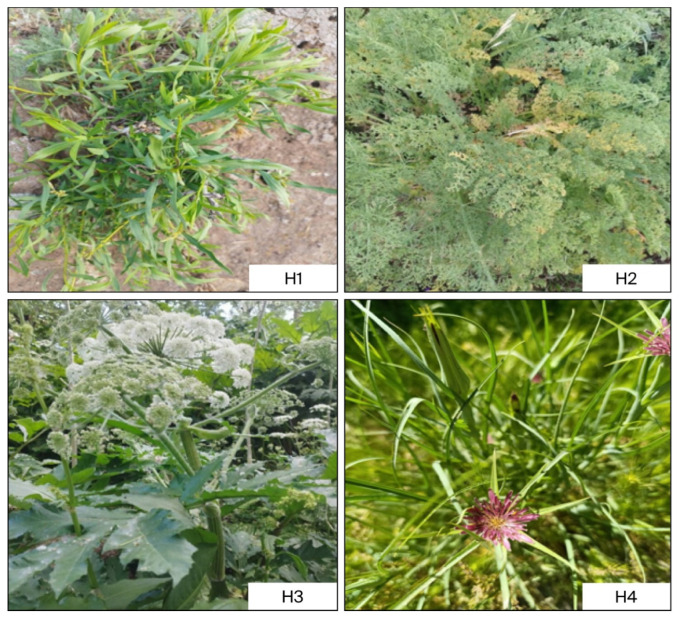
The plant samples. H1; *Daphne mucronata*, H2; *Ferula communis*, H3; *Heracleum persicum*, H4; *Tragopogon coloratus*.

*Daphne mucronata* (labelled as H1), *Ferula communis* (labelled as H2), *Heracleum persicum* (labelled as H3), and *Tragopogon coloratus* (labelled as H4) used in this study were collected in May and June 2024 from Bağdaş village, Kayakonak hamlet, in Yüksekova district of Hakkari province, located at coordinates 37.65° N and 44.26° E in southeastern Turkey. The leaf parts of all plant species were used in the analyses, as they are traditionally consumed and preferred in local ethnomedicinal practices. The plant samples (Figure 2) brought to the laboratory were dried in the dark at room temperature for 15 days, as is done by the local people. All samples were ground in the laboratory using a plant grinder (IKA A 11 basic, IKA-Werke GmbH & Co. KG, Staufen, Germany). The powdered samples were freeze-dried in a lyophilization device at −79 °C and 0.04 mbar for 36 h to remove moisture, then stored at −18 °C for further analyses.

### 2.4. Heavy Metal and Mineral Analyses Using ICP-OES (Inductively Coupled Plasma Optical Emission Spectroscopy) and AAS (Atomic Absorption Spectroscopy)

The Milestone Ethos Easy Microwave digestion system (Milestone Srl, Sorisole, Bergamo, Italy) was employed to dissolve 200 milligrams of lyophilized plant material in Teflon tubes at 195 °C and 36 bar pressure for 50 min. The composition comprised two millilitres of hydrogen peroxide and six millilitres of nitric acid. The plant samples were subjected to analysis via atomic absorption spectrometry (AAS-ICE 3000 series, Thermo Fisher Scientific, Waltham, MA, USA) and inductively coupled plasma optical emission spectrometry (ICP-OES, iCAP 6000 series, Thermo Fisher Scientific, Waltham, MA, USA) following solubilization by the Milestone Ethos Easy Microwave Digestion System (microwave wet digestion), as per the literature [33]. The ICP-OES was used to measure the concentrations of aluminium (Al), arsenic (As), cadmium (Cd), cobalt (Co), chromium (Cr), copper (Cu), iron (Fe), nickel (Ni), lead (Pb), and zinc (Zn). The concentrations of calcium (Ca), potassium (K), magnesium (Mg), and sodium (Na) were measured using Atomic Absorption Spectroscopy (AAS) devices. Three separate analyses were conducted. The concentration of each element is shown in milligrams per kilogram of dry weight. To ensure the accuracy and precision of ICP-OES and AAS, method validation (Table 1 and Table 2) was performed using NIST SRM 1570a spinach leaves (National Institute of Standards and Technology, Gaithersburg, MD, USA), a validated reference material, and considering traditional plant matrix acceptability criteria [34], to determine the limit of detection (LOD) and limit of quantification (LOQ).

The signal-to-noise ratio analysis by ICP–OES determined the detection and quantification limits, which were set at three times the baseline noise for LOD and ten times for LOQ (Table 1). The precision of the results was demonstrated by relative standard deviation (%RSD) values that ranged from 0.95% to 1.55%, indicating high repeatability. The calibration curves for all analytes demonstrated exceptional linearity because their R^2^ values exceeded 0.9996. The analytical method demonstrated reliable heavy metal quantification in plant matrices, as recovery tests with CRM samples yielded results between 90% and 96%.

Using verified reference material (NIST SRM 1570a) to ensure the accuracy of atomic absorption spectrometry (AAS) measurements, validation of the method was conducted for calcium, potassium, magnesium, and sodium. Signal-to-noise ratios were employed to establish the limits of detection and quantification. The precision of the measurements, expressed as percent relative standard deviation (%RSD), consistently remained below 1.2%, indicating excellent repeatability (refer to Table 2). The calibration curves demonstrated strong linearity (R^2^ > 0.999), with recovery rates ranging from 90% to 92.5%, thereby confirming that the analytical method accurately and reliably quantifies macroelements in plant matrices.

### 2.5. Health Risks Associated with Heavy Metals from Infusions of Traditional Medicinal Plants in Hakkâri, Türkiye

The potential health impacts of heavy metal exposure from infusions of *Daphne mucronata*, *Ferula communis*, *Heracleum persicum*, and *Tragopogon coloratus* were assessed using a human health risk assessment protocol. Transfer factors for arsenic (100%), cadmium (6.6%), chromium (42%), and lead (19.8%) were derived from peer-reviewed studies on herbal infusions. Due to the absence of valid infusion transfer data in the existing literature for cobalt, copper, iron, zinc, nickel, and aluminum, these elements were excluded from the calculations of Environmental Distribution Coefficient (EDC), Health Index (HI), and Cancer Risk (CR) to avoid speculative assumptions.

The methodology adhered to international guidelines [34], and to ensure clarity and consistency throughout the study, previously reported [35] variable abbreviations were maintained:

Estimated Daily Consumption (EDC)

The estimated daily intake (EDC) for each hazardous metal was calculated using the following modified Equation (1):

(1) $${EDC}_{a}=\frac{M_{sw}\times D_{i}\times T_{s}}{W_{b}}$$

*M_sw_* = the amount of metal in the dried plant material (mg/kg),

*D_i_* = daily intake of tea (0.0114 kg/day [36]),

*T_s_* = the percentage of metal that moves from the plant into tea during brewing (%),

*W_b_* = average adult body weight (68 kg, approximately the weight for Turkish people).

The transfer percentages were determined from research investigating the migration of elements from tea and botanical matrices into hot water [36,37,38]: As = 100%, Pb = 19.8%, Cr = 42%, and Cd = 6.6%.

Target Hazard Level (THL)

The target hazard rate (THL) was used to figure out the non-carcinogenic health risk for each element (2). According to values documented in the literature [35,38], the reference dosages (RfDa) utilized in this investigation were 1.0 × 10^−4^ mg/kg/day for Cd, 1.5 × 10^−3^ mg/kg/day for Cr, 3.6 × 10^−3^ mg/kg/day for Pb, and 3.1 × 10^−4^ mg/kg/day for As.

A Target Hazard Level (THL) over 1 was considered indicative of possible non-carcinogenic health impacts.

(2) $${THL}_{a}=\frac{{EDI}_{a}}{{RfD}_{a}},$$

Hazard Index (HI)

To understand how different metals might act together, we used the Hazard Index (HI), which adds up the Target Hazard Level (THL) values calculated for each element (3):

(3) $$HI=\sum {THQ}_{x}$$

Exposure to many contaminants may pose a health concern if the HI is more than 1. Many metals can combine to generate issues unrelated to cancer. In this formula, “a” stands for each heavy metal that was measured in the samples. The sigma sign (∑) implies that all the THL values that matter are added together to generate a single number that shows the total risk. Only elements with measured concentrations and an accessible reference dosage (RfD) were included, ensuring that the ranking is based on accurate measurement data.

Cancer Risk (CR)

Some heavy metals, including cadmium (Cd), arsenic (As), and hexavalent chromium (Cr VI), can raise the risk of getting cancer throughout a lifetime. To calculate this potential (4), we multiplied the estimated daily intake (EDC) of each element by its cancer slope factor (CSF):

(4) $${CR}_{a}={EDC}_{a}\times {CSF}_{a}$$

The CSF values utilized were: 6.1 mg kg^−1^ day^−1^ for Cd, 1.5 mg kg^−1^ day^−1^ for As, and 0.5 mg kg^−1^ day^−1^ for Cr(VI). Lead (Pb) was not included in this computation because there is no official CSF for it from the USEPA or ATSDR (Agency for Toxic Substances and Disease Registry), even though IARC (Inorganic and Organic Lead Compounds) [39] classifies lead as “probably carcinogenic to humans”. As a result, Pb was tested exclusively for non-cancer effects with THL and HI. The US Environmental Protection Agency (US EPA) says that exposure is too high for public health when the total CR is between 1 × 10^−4^ and 1 × 10^−6^ [40].

### 2.6. Applications of Scanning Electron Microscopy with Energy-Dispersive X-Ray Spectroscopy (SEM-EDX) for Surface Morphology and Elemental Composition Analysis

The surface microstructure of the *Daphne mucronata* (H1) root was examined using a Zeiss Sigma 300 field-emission scanning electron microscope (FE-SEM). The microscope operated at an accelerating voltage of 10 kV, and micrographs were collected at multiple magnifications, focusing on regions corresponding to the highest elemental levels previously indicated by ICP^−^OES results. The elemental distribution and composition of the examined areas were further characterized through an Ametek EDAX energy-dispersive X–ray (EDX) detector integrated into the FE-SEM unit. SEM-EDX analysis was conducted on all four species; however, only the root surface image of *Daphne mucronata* was included in the manuscript due to significantly high levels of heavy metal accumulation. This decision was made to visually support the elemental data and highlight metal uptake mechanisms in the most affected species.

### 2.7. Antioxidant Capacity Analyses

Because each antioxidant assay responds differently to the chemical nature of compounds, DPPH, ABTS, and CUPRAC assays were applied together to capture a broader and more accurate picture of the extracts’ free-radical scavenging potential.

#### 2.7.1. DPPH and ABTS Analyses

The DPPH (2,2-diphenyl-1-picrylhydrazyl) assay was modified using the previously reported procedures [33,41]. A 0.2% (*m*/*v*) DPPH solution was prepared in ethanol and stored in the dark until needed. The test samples were adjusted to different concentrations, and 3 mL of the radical solution was mixed with an equal volume of each sample. To ensure optimal interaction, the mixes were held at room temperature (25 °C) in the dark for 40 min, shaking continuously. All determinations were made in triplicate. The vials were kept dark during the incubation period. Thereafter, the absorbance at 517 nm was measured using a Multiskan SkyHigh microplate spectrophotometer (Thermo Fisher Scientific, Waltham, MA, USA) with ethanol acting as the blank. The proportion of DPPH radical scavenging was calculated using the following equation [33].

*DPPH Radical Scavenging Capacity (%) = [(Ax − Az) ÷ Ax] × 100*

where Ax is the DPPH solution’s absorbance and Az is the amount of light that the mixture of sample and DPPH absorbs.

The extracts’ antioxidant capacity was also assessed using the ABTS assay, which was carried out in accordance with the protocol outlined in previous research [33,42]. This method assesses an antioxidant’s ability to quench the ABTS•^+^ radical cation (2,2′-azinobis-(3-ethylbenzothiazoline-6-sulfonic acid)). 2.5 mM potassium persulfate and 2 mM ABTS were mixed, and the mixture was left at room temperature in the dark for 12 h to produce the radical. Before analysis, the solution was diluted with ethanol until its absorbance at 734 nm was 0.72 ± 0.02. Plant extracts were prepared at concentrations of 10, 25, 50, and 100 mg/mL for the test. 200 µL of the ABTS•^+^ solution was mixed with a 20 µL aliquot of each extract, and the reaction mixtures were then incubated in the dark for 25 min. Using a Multiskan SkyHigh microplate spectrophotometer (Thermo Fisher Scientific, USA), absorbance readings were taken at 734 nm after the samples had been incubated. Using the same formula as in the DPPH assay, the percentage of ABTS radical scavenging was calculated. Both assays demonstrated the extracts’ ability to combat free radicals by preventing their formation. Butylated hydroxytoluene (BHT) was used as the reference antioxidant.

#### 2.7.2. CUPRAC Assay for Antioxidant Determination

Extracts’ antioxidant potential was measured at four concentrations using the CUPRAC (cupric reducing antioxidant capacity) assay [43]. Extracts and standards were mixed with Cu(II), neocuproine, and ammonium acetate buffer, with final concentrations of 20, 40, 80, and 100 µg/mL. A 450 nm absorbance was measured after 65 min of mixing. Sample readings were compared to reference standards. All tested extracts were compared to BHT and α-tocopherol as standards, and the total antioxidant capacity was measured using the TEAC (µg/mL).

### 2.8. Sample Preparation for HPLC and Antioxidant Evaluation

The extraction procedure was followed as reported in a previous study [33]. Lyophilized samples were mixed with 80% acidified ethanol (*v*/*v*/*v*; EtOH 80.0%, HCl 0.1%, H_2_O 19.9%) at a ratio of 2:20 (*w*/*v*) and incubated at 23 ± 1 °C for three hours. The homogenized plant material was subjected to centrifugation at 20,000× *g* (≈15,000 rpm) for 20 min at 3 °C using a Hitachi CR22N refrigerated centrifuge (Hitachi Koki Co., Ltd., Tokyo, Japan) to separate particulate residues. The resulting supernatant was collected and concentrated under vacuum at 38 °C (110 rpm, 70 min) using a Heidolph Laborota 4000 rotary evaporator (Heidolph Instruments GmbH & Co. KG, Schwabach, Germany) to remove ethanol. The viscous concentrate obtained was subsequently lyophilized in a LyoQuest-Telstar freeze-dryer (Telstar, Terrassa, Spain) at –79 °C (0.05 psi, 70 h) to ensure complete dehydration. The dried extract was dissolved in ethanol (1 mg/mL), filtered through 0.45 µm PTFE membranes, and stored at 4 °C for later chromatographic and antioxidant analyses.

#### Chromatographic Parameters for HPLC Analysis of Flavonoid and Phenolic Compounds

**Figure 3:**
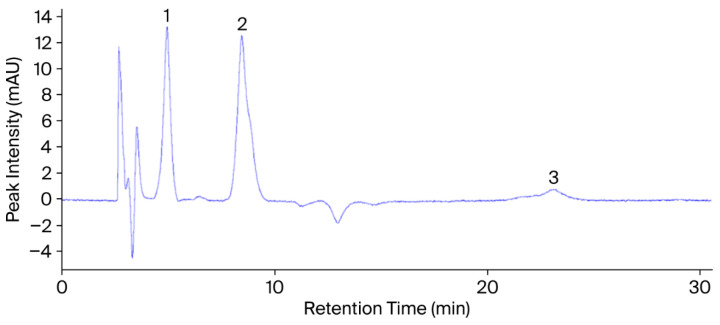
HPLC chromatogram of phenolic and flavonoid standards obtained at 254 nm under the specified chromatographic conditions. Peaks: (1) protocatechuic acid (tR = 4.90 min), (2) vanillic acid (tR = 8.42 min), (3) rutin (tR = 23.32 min). Unlabeled peaks correspond to other phenolic standards analyzed in the same batch as part of a broader reference library and are not related to the compounds quantified in the present study.

A diode array detector (DAD) operating at 254 nm and 280 nm was integrated into a Thermo Finnigan Surveyor HPLC system (Thermo Fisher Scientific Inc., Waltham, MA, USA). Figure 2 and Figure 3 present the chromatograms and retention times of the phenolic and flavonoid (rutin) standards. An Agilent Zorbax Eclipse XDB-C18 column (4.6 × 250 mm, 5 µm; Agilent Technologies Inc., Santa Clara, CA, USA) maintained at 40 °C was employed for separation. The system consisted of an SCL-10A controller, a DGU-12A degasser, an LC-10ADVP pump, and a Surveyor autosampler (Shimadzu Corp., Kyoto, Japan).

Chromatographic separation was performed under isocratic conditions using a mobile phase composed of methanol (29%), acetic acid (3%), and water (68%) (*v*/*v*/*v*). The solvent composition remained constant throughout the run. At the same time, the flow rate was gradually adjusted—from 1.0 mL min^−1^ initially to 1.30 mL min^−1^ at 10 min and 1.35 mL min^−1^ at 16 min—to enhance resolution and improve peak symmetry for late-eluting compounds. Although this adjustment did not constitute a compositional gradient, it was optimized through preliminary trials to achieve better separation efficiency. The total run time was 30 min, during which all compounds were fully eluted under these conditions.

**Figure 4:**
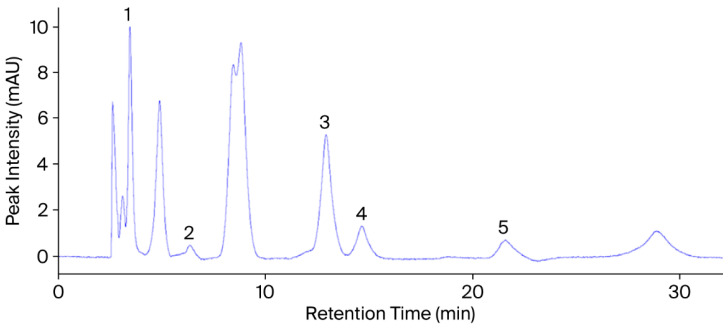
HPLC chromatogram of phenolic standards obtained at 280 nm under the specified chromatographic conditions. Peaks: (1) gallic acid (tR = 3.47 min), (2) chlorogenic acid (tR = 6.35 min), (3) p-coumaric acid (tR = 12.92 min), (4) ferulic acid (tR = 14.65 min), (5) o-coumaric acid (tR = 21.59 min). Unlabeled peaks correspond to other phenolic standards analyzed in the same batch as part of a broader reference library and are not related to the compounds quantified in the present study.

The injection volume was 20 µL. Before injection, standards and extracts were dissolved in HPLC-grade methanol and filtered through 0.45 µm membrane filters. Each compound was identified by comparing its retention time (tR) with that of the corresponding reference standard (Figure 3 and Figure 4). Quantification was carried out using an external standard calibration approach. Standard solutions were prepared at five concentration levels, and calibration curves were obtained by plotting peak area (mAU·s) against concentration (µg mL^−1^). Each analyte concentration in the plant extracts was calculated using its respective regression equation. Rather than optimizing individual detection wavelengths for each compound, a diode array detector (DAD) was used for dual-wavelength monitoring at 254 and 280 nm, facilitating the practical detection of multiple phenolic compounds. Although complete spectral data were not digitally recorded, the UV-Vis spectra of eluted peaks were visually monitored in real-time and found to be consistent with those of the standards. Compound identification was based on both retention time (tR) and qualitative spectral match.

### 2.9. Statistical Analysis

All experimental data are expressed as mean ± standard deviation (SD) based on three independent replicates. Prior to interspecies comparison, the dataset was examined to confirm compliance with the assumptions of normality and homogeneity of variance. When these prerequisites were satisfied, differences among plant groups were evaluated using one-way analysis of variance (ANOVA) followed by Tukey’s post hoc test. For datasets violating these assumptions, a Kruskal–Wallis test supplemented with Dunn’s multiple comparison adjustment was employed. Risk-related indices were summarized descriptively. All statistical computations were carried out in IBM SPSS Statistics version 25, and statistical significance was defined at a *p*-value < 0.05 threshold.

## 3. Results and Discussion

### 3.1. Levels of Heavy Metals and Essential Minerals in Samples

Inductively coupled plasma optical emission spectrometry (ICP-OES) analysis showed that the four medicinal plants from Hakkari, Türkiye, *Daphne mucronata* (H1), *Ferula communis* (H2), *Heracleum persicum* (H3), and *Tragopogon coloratus* (H4), had different amounts of heavy metals (Table 3). H1 had the highest levels of all the elements, and these levels were often higher than those considered safe according to international standards. H2-H4, on the other hand, was mostly within the safe levels.

There was a large difference in chromium levels (*p* < 0.001). H1 (2.85 ± 0.20 mg/kg) was above the herbal material limit of 2 mg/kg, but the other species stayed below 0.7 mg/kg. The arsenic level in H1 (0.78 ± 0.05 mg/kg) was also higher than the maximum level of 0.6 mg/kg. The levels in the other taxa, on the other hand, were much lower (0.18–0.22 mg/kg). The amount of lead was about the same: H1 had 14.6 ± 0.6 mg/kg, which is more than the 10 mg/kg limit, and H2–H4 had about 1.2–1.5 mg/kg. H1 had slightly higher levels of copper and zinc than the recommended amounts, which are 34.2 and 31.5 mg/kg, respectively. In all species, Cd, Co, Fe, Ni, and Al stayed within normal or acceptable ranges. In contrast, H1 again exhibited the highest mean values.

These findings indicate that *Daphne mucronata* is an efficient accumulator of Pb, As, Cr, Cu, and Zn. Turkish medicinal plants have been shown to have a similar deposition system, which is affected by the location and plant species. For example, Karahan et al. (2020) [44] found Pb levels as high as 16 mg/kg and Cr levels as high as 42 mg/kg in ethnobotanical species from southern Türkiye; however, most species remained below the WHO/FAO limits. Nationwide surveys indicate that Pb and Cr levels are generally safe on average, but they may increase in areas where mining or heavy traffic is present [21,45].

The variability is similar when looked at from a global perspective. Kandić et al. (2023) [46] found that Serbian medicinal plants had Pb levels ranging from 0.6 to 49 mg/kg and Ni levels up to 12 mg/kg. Some of these levels exceeded the WHO limits, similar to what was found for H1. Market samples of *Heracleum persicum* in Iran frequently displayed lead (30–33 mg/kg) and cadmium (5–11 mg/kg) concentrations exceeding permissible limits [47]. A global review found that a significant number of herbal products still contain Pb, As, and Cd, highlighting the importance of monitoring quality [20,48].

The high levels of Pb, As, and Cr in D. mucronata could be attributed to geological enrichment, past mining, atmospheric deposition, and the plant’s ability to move and recycle metals under specific pH and organic matter conditions [49,50,51]. In terms of public health, the high concentrations found in *D. mucronata* suggest that raw material from contaminated habitats should not be consumed without prior screening.

**Table 3:** Heavy metal concentrations (mg/kg) in four medicinal plants from Hakkari, Türkiye, determined by ICP-OES and compared to worldwide safety standards.

Element	Guideline/Limit (mg/kg, Dry)	H1	H2	H3	H4	*p*-Value	References
Cr	not exceeding: 2 mg/kg	2.85 ± 0.20 ^c^	0.62 ± 0.04 ^b^	0.45 ± 0.03 ^a^	0.51 ± 0.04 ^ab^	0.0007	[49,52]
As	not exceeding: 0.6 mg/kg	0.78 ± 0.05 ^c^	0.22 ± 0.02 ^b^	0.18 ± 0.01 ^ab^	0.21 ± 0.02 ^b^	0.012	[53]
Cd	not exceeding: 0.3 mg/kg	0.24 ± 0.02 ^c^	0.08 ± 0.01 ^b^	0.06 ± 0.01 ^b^	0.07 ± 0.01 ^b^	0.018	[53]
Co	not exceeding: 0.5 mg/kg	0.42 ± 0.03 ^c^	0.19 ± 0.02 ^b^	0.16 ± 0.02 ^b^	0.17 ± 0.02 ^b^	0.001	[49]
Cu	not exceeding: 30 mg/kg	34.2 ± 1.4 ^c^	7.3 ± 0.4 ^a^	9.1 ± 0.5 ^b^	8.5 ± 0.5 ^b^	0.0009	[49,54]
Fe	not exceeding: 250 mg/kg	175.5 ± 5.1 ^c^	54.3 ± 2.2 ^a^	61.2 ± 2.5 ^ab^	58.9 ± 2.4 ^ab^	0.0013	[49,54]
Pb	not exceeding: 10 mg/kg	14.6 ± 0.6 ^c^	1.2 ± 0.1 ^a^	1.5 ± 0.1 ^a^	1.3 ± 0.1 ^a^	0.0001	[53,55]
Zn	not exceeding: 27 mg/kg	31.5 ± 1.2 ^c^	10.8 ± 0.4 ^a^	12.1 ± 0.5 ^a^	11.5 ± 0.4 ^a^	0.0003	[49,54]
Ni *	no ML; EFSA TDI 2.8 μg/kg bw/day	1.8 ± 0.08 ^c^	0.48 ± 0.03 ^b^	0.42 ± 0.02 ^b^	0.45 ± 0.02 ^b^	0.0004	[49,56]
Al *	no ML; typical background 1–15 mg/kg	12.5 ± 0.6 ^c^	4.1 ± 0.3 ^b^	3.8 ± 0.2 ^b^	3.6 ± 0.3 ^b^	0.001	[49]

Values are expressed as mean ± standard deviation (n = 3). Superscript letters (a–c) indicate statistically significant differences (*p* < 0.05). H1—*Daphne mucronata*; H2—*Ferula communis*; H3—*Heracleum persicum*; H4—*Tragopogon coloratus*; ML—maximum level; TDI—tolerable daily intake; SD—standard deviation; bw—body weight. * Ni and Al: no formal maximum level (ML); evaluation based on EFSA TDI (Ni) or background concentrations (Al).

The concentrations of four essential macro-minerals, magnesium (Mg), calcium (Ca), sodium (Na), and potassium (K), were quantified in the leaves of *Daphne mucronata* (H1), *Ferula communis* (H2), *Heracleum persicum* (H3), and *Tragopogon coloratus* (H4) using atomic absorption spectroscopy (AAS), as shown in Table 4.

Magnesium concentrations varied noticeably among the species. The highest level was detected in H2 (147.5 ± 4.8 mg kg^−1^), whereas H1 showed a slightly lower but still substantial value. H3 (93.2 ± 6.3 mg kg^−1^) and H4 (86.4 ± 3.9 mg kg^−1^) contained the least magnesium, and the overall variation was statistically significant (*p* = 0.0008). Calcium was abundant in all species but especially in H1 (502.5 ± 10.3 mg kg^−1^), which exceeded the values recorded for H3 and H4 by more than 60%. Even the least-rich sample, H4 (289.4 ± 6.2 mg kg^−1^), remained within the lower end of internationally recommended limits. Sodium concentrations were comparatively modest, ranging between 28.4 ± 1.7 and 52.0 ± 2.1 mg kg^−1^. Although the interval was narrow relative to other minerals, the variation among plants was still significant (*p* = 0.0013). Potassium exhibited the widest amplitude of all measured elements. The leaves of H1 contained an exceptionally high amount (715.2 ± 9.6 mg kg^−1^), whereas H4 accumulated only 108.4 ± 4.7 mg kg^−1^, resulting in a more than sixfold difference across species (*p* = 0.0001).

In general, the macro-mineral profiles of the plants that were analyzed were within or below the ranges that had been reported as acceptable for culinary or medicinal herbs in previous studies. In Türkiye, Özcan (2004) [57] found that many condiment/herb species had Ca, K, and Mg levels in similar orders of magnitude but often higher values where the fertility of the soil or conditions for growth were favorable. Another Turkish study on wild edible plants found K levels as high as ~2600 mg per 100 g dry weight (≈26,000 mg/kg) for some species. This is significantly higher than what we observe in these medicinal plants, indicating that H1–H4 are moderate in comparison [58]. Studies conducted outside of Türkiye, such as those on Moroccan medicinal and aromatic plants, found similar broad ranges of Ca, Mg, and K among species, with comparable statistical differences [59].

**Table 4:** Mineral content (mg/kg) of four medicinal plant samples analyzed by AAS (compared with international limits and literature references).

Mineral (Limit, mg/kg)	H1	H2	H3	H4	*p*-Value	References
Magnesium (1000–5000)	124.5 ± 5.5 ^a^	147.5 ± 4.8 ^b^	93.2 ± 6.3 ^c^	86.4 ± 3.9 ^c^	0.0008	[60]
Calcium (2000–10,000)	502.5 ± 10.3 ^a^	487.0 ± 9.8 ^b^	315.5 ± 7.4 ^c^	289.4 ± 6.2 ^c^	0.0004	[54,60]
Sodium (100–2000)	52.0 ± 2.1 ^a^	44.3 ± 1.9 ^b^	31.5 ± 2.5 ^c^	28.4 ± 1.7 ^c^	0.0013	[53]
Potassium (10,000–30,000)	715.2 ± 9.6 ^a^	275.1 ± 8.4 ^b^	193.8 ± 6.1 ^c^	108.4 ± 4.7 ^d^	0.0001	[54,60]

Values are expressed as mean ± standard deviation (n = 3). Superscript letters (a–d) indicate statistically significant differences (*p* < 0.05). H1: *Daphne mucronata;* H2: *Ferula communis;* H3: *Heracleum persicum;* H4: *Tragopogon coloratus.*

### 3.2. Heavy Metal Related Health Risks from Infused Traditional Medicinal Plants in Hakkâri (Turkey)

Risk estimations were carried out solely for As, Cd, Cr, and Pb, which had approved transfer percentages from plant to infusion (Table 5 and Table 6). There was no consistent transfer data for Co, Cu, Fe, Zn, Ni, and Al; hence, the results for these elements are not reported, and more research is recommended.

As shown in Table 5, the hazard index (HI) for *Daphne mucronata* (H1) was 0.64, indicating a combined impact of arsenic (THL = 0.436) and lead (THL = 0.138). Chromium and cadmium contributed modestly (THL = 0.0669 and 0.0027, respectively). Although HI remained below the standard value of one, H1 exhibited the highest potential non-carcinogenic risk of the plants evaluated. *Ferula communis* (H2), *Heracleum persicum* (H3), and *Tragopogon coloratus* (H4) exhibited HI values of 0.15, 0.13, and 0.14, respectively, which were all within commonly accepted ranges. Table 6 also shows that H1 had the highest lifetime cancer risk (CR) of 4.25 × 10^−3^. The majority of the risk (4.18 × 10^−3^) was due to arsenic, whereas cadmium (1.62 × 10^−5^) and chromium (5.42 × 10^−5^) had minor roles. The risks for the other plants were much lower: 1.20 × 10^−3^ for H2 (*Ferula communis*), 9.78 × 10^−4^ for H3 (*Heracleum persicum*), and 1.14 × 10^−3^ for H4 (*Tragopogon coloratus*). All estimates are higher than the U.S. EPA’s standard of 1 × 10^−6^, but they are within the highest range found for some herbal infusions in low-exposure situations [46].

The elevated HI and CR reported for *Daphne mucronata* indicate that, under extreme conditions (100% transfer, 100% inorganic As, 11.4 g/day ingestion), this plant may represent a health risk if ingested daily over extended durations. Similar results have been observed in certain teas and medicinal herbs, where arsenic concentrations predominated in the total risk assessment [60]. Conversely, the remaining three plants exhibited CR values within or slightly beyond the 10^−4^ management range, consistent with prior studies on heavy metal exposure via infusions [61,62].

Arsenic was the leading cause of cancer risk in all samples, whereas cadmium and chromium were less important. This finding is similar to that of Speer et al. (2023), who identified arsenic as the primary cause of cancer risk in food [63]. It is essential to note that all non-cancer indices remained far below one, indicating that a moderate consumption of these infusions is unlikely to have negative consequences. Not only that, but the fact that H1 is close to the upper end of reported ranges means that regular consumption should be monitored, especially for groups that are more likely to be affected. Future studies that look into inorganic As speciation and realistic ingestion situations would help us obtain better estimations of the risks and make sure that these plants can be safely used in traditional medicine.

The carcinogenic risk (CR) for arsenic, chromium, and cadmium in four therapeutic plant infusions is shown in Table 6. The total CR for *Daphne mucronata* (H1) is 4.25 × 10^−3^, with arsenic alone making up 4.18 × 10^−3^ of that. Chromium and cadmium, on the other hand, make up much smaller amounts, 5.42 × 10^−5^ and 1.62 × 10^−5^, respectively. The total CR values for the other plants are 1.20 × 10^−3^ for *Ferula communis* (H2), 9.78 × 10^−4^ for *Heracleum persicum* (H3), and 1.14 × 10^−3^ for *Tragopogon coloratus* (H4). All of these are higher than the commonly used acceptable level of 1 × 10^−6^, and many of them are also higher than the less strict upper limit of 1 × 10^−4^ used in risk management situations. Compared to other studies, the CR values for H2 and H4 are high. In a review of 227 tea and herbal infusion studies, Hu et al. (2023) reported that certain samples’ CR values approached 10^−3^–10^− 2^ for arsenic in teas with high contamination or intake levels [64]. In research on medicinal plants marketed in Ado Ekiti, Olusola et al. (2021) found elevated arsenic risk indices in some samples, with CR estimations exceeding 10^−3^ in the worst cases [65]. However, reasonable consumption assumptions resulted in lower levels. Many global studies have found substantially lower CR values. For example, black tea samples from Assam and North Bengal revealed a hazard quotient <1 and CR less than 10^−4^ under average consumption and transfer rates [66]. Additionally, a thorough investigation into herbal and traditional teas was conducted by Oliveira et al. (2018) [67]. The researchers discovered that while certain herbal teas did have total arsenic levels that were higher than the recommended limits, the resulting CR under standard intake rarely went beyond 10^−4^ when more practical factors for transfer and speciation were taken into account [67]. Even though the numbers for H2 and H4 are high, they are just above what has been seen in the literature under similar severe assumptions. These data highlight the fact that arsenic remains the primary contributor to carcinogenic risk in herbal infusions under worst-case situations.

### 3.3. Scanning Electron Microscopy with Energy-Dispersive X-Ray Spectroscopy (SEM-EDX) of H1 Sample

**Figure 5:**
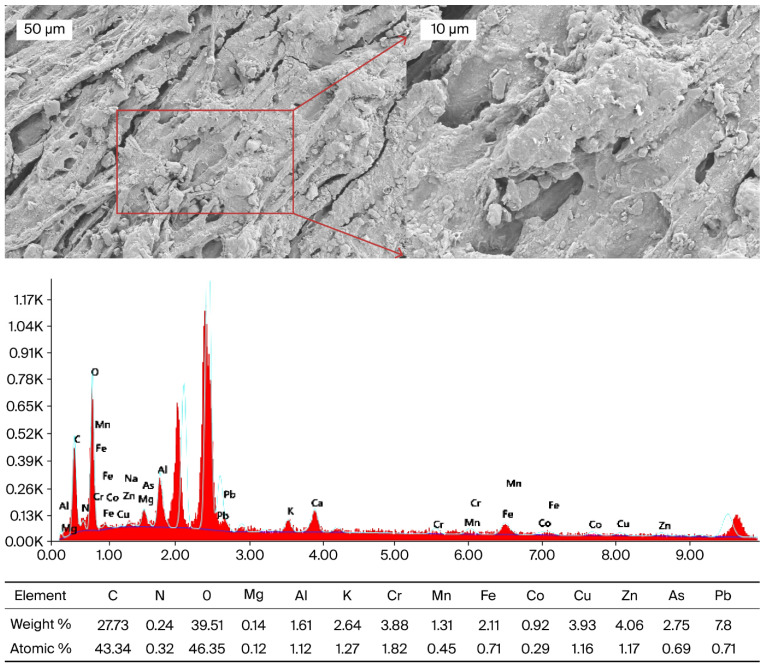
SEM-EDX analysis of *Daphne mucronata* (H1) dried root sample.

In this study, SEM-EDX was applied to morphologically demonstrate the distribution of heavy metals in the dried root sample of *Daphne mucronata* (H1) (Figure 5) and to support ICP-OES data. ICP-OES analysis revealed high levels of Pb, Cr, Cu, Zn and As in *Daphne mucronata* (H1) dried leaf samples. However, the entry and accumulation sites of heavy metals in plants are predominantly root tissues. Therefore, SEM-EDX analysis was performed on the roots to show the starting points of metal uptake and the possible sources of high metal content in the leaves. Similarly, in the literature, SEM-EDX analyses performed on root surfaces have been reported to successfully reveal the retention regions of elements such as cadmium, arsenic, and uranium [68,69,70]. An irregular and fibrous surface structure was observed in the SEM image of the root tissue. Small voids are considered to be active regions where metal ions can bind. EDX spectra showed that the surface consists mainly of O (39.51%), C (27.73%) and N (0.24%), while Mg, Al, K, Cr, Mn, Fe, Co, Cu, Zn, As and Pb elements are present in trace or higher proportions. The particularly high levels of Pb (7.80%) and As (2.75%) indicate that these elements form a layer on the root surface together with Cu (3.93%), Zn (4.06%), and Cr (3.88%). These results are consistent with ICP-OES data and support the notion that the high levels of Pb, Cr, Cu, Zn, and As in the leaves are related to accumulation in the roots.

The findings indicate that the root tissue of *Daphne mucronata* plays a critical role in heavy metal uptake. Phenolic, hydroxyl, and carboxyl functional groups in the lignocellulosic structure may facilitate binding to the root surface by chelating Pb^2+^ and As oxo-anions [71]. Similarly, previous studies have indicated that Pb and As are predominantly retained in the root systems of plants growing in arid and semi-arid regions [72]. He et al. (2024) also emphasised that SEM-EDX is a powerful technique that can show where metals accumulate and how they are distributed in plant tissues at high resolution [73]. Future research should include comparative SEM-EDX mapping of root, stem, and leaf tissues, supported by Micro X-ray Fluorescence (µXRF) and Laser Ablation-Inductively Coupled Plasma Mass Spectrometry (LA-ICP-MS) methods, which could reveal in greater detail how metals are distributed within the plant tissues and in which regions they are concentrated.

The four plant species examined in this study have been traditionally used for medicinal purposes by local communities in southeastern Türkiye, particularly in the Hakkâri region, for centuries. This research goes beyond a scientific investigation, aiming to bridge traditional ethnobotanical knowledge with modern pharmacological science. Considering their bioactive potential, identifying toxic element accumulation is an important step to ensure the safe use of these species in both traditional and modern medicine. The results of this study may help evaluate local plants as potential natural sources for future drug development and support further research on regional plant diversity and pharmaceutical safety.

### 3.4. Phenolic Acid Results (Via HPLC) and Antioxidant Capacities of Samples

High-pressure liquid chromatography (HPLC) is presently the most prevalent and dependable technique for the identification and quantification of flavonoid and phenolic compounds. This approach allows for the utilization of various columns and mobile phases according to the polarity and chemical characteristics of the analytes. Isocratic and gradient elution methods can be utilized to get optimum compound separation. In this study, isocratic elution was applied because it provides higher reproducibility, stable baseline conditions, and consistent peak resolution for flavonoid and phenolic compounds under fixed mobile phase composition. Since the phenolic composition of the plant extracts was not highly complex, an isocratic system allowed sufficient separation efficiency without the need for a gradient program, minimizing solvent variation and improving quantification accuracy. Similar methodological approaches have been reported in HPLC analyses of plant phenolics, where isocratic elution was preferred for stable peak resolution and reliable baseline separation of simple phenolic profiles [74,75,76].

The high extraction efficiency obtained in this study can be attributed to the use of 80% acidified ethanol (EtOH 80.0%, HCl 0.1%, H_2_O 19.9%), which facilitates the hydrolysis of ester and glycosidic bonds and enhances the release of bound phenolic acids and flavonoid glycosides. These results are consistent with previous reports, where acidified ethanol systems effectively improved phenolic and flavonoid extraction from various plant matrices, including leafy vegetables, seeds, and edible flowers [76,77,78]. The influence of solvent polarity and extraction parameters on phenolic recovery has also been highlighted by Azwanida [79], supporting the selection of this solvent system in our work.

HPLC-DAD can detect compounds in plant extracts by comparing their retention periods and UV-visible absorption spectra to established standards. All phenolic compounds have significant absorption in the ultraviolet range, with each class of phenolics exhibiting distinct absorption peaks. For example, ellagitannins typically absorb at roughly 250 nm, while hydroxybenzoic acids, isoflavones, flavanones, and catechins absorb at 280 nm. In contrast, hydroxycinnamic acids, stilbenes, and flavones exhibit peak absorption near 320 nm.

In this study, flavonoid and phenolic compounds were identified by comparing their retention periods and UV spectra to those of reference standards. Quantification was executed utilizing the external standard approach, predicated on calibration curves developed for each standard chemical. The concentrations of different phenolics and flavonoid (rutin) were reported as milligrams per 100 g of dry weight (mg/100 g DW) of the extracts.

Table 7 shows that the four species (*Daphne mucronata*, *Ferula communis*, *Heracleum persicum*, *and Tragopogon coloratus)* have quite different flavonoid (rutin) and phenolic acid profiles. *Daphne mucronata* (H1) has the most gallic acid (5.20 mg/g), followed by *Heracleum persicum* (H3). *Ferula communis* (H2) and *Tragopogon coloratus* (H4) had much lower amounts. Chlorogenic acid was most plentiful in H4, with a concentration of 7.80 mg/g. H2 and H3 had high quantities of p-coumaric and ferulic acids, while H3 had the most rutin, and H4 had the least. The order of protocatechuic and vanillic acids was H3 > H1 > H2 > H4. These differences are statistically significant (ANOVA + Tukey, *p* < 0.05) and match the letters that are above the numbers in the table. The highest levels of gallic acid were found in H1 (*Daphne mucronata*), matching HPLC studies that found gallic acid and rutin among flavonoids in *D. mucronata* leaves [2]. As previously observed [80], H2 (*Ferula communis*) had significant amounts of p–coumaric, o–coumaric and ferulic acids, as well as chlorogenic acid. H3 (*Heracleum persicum*) contained high levels of vanillic, ferulic, and chlorogenic acids, which were identified as important compounds across multiple growth stages in a phenological study [81]. H4 (*Tragopogon coloratus*) was dominated by chlorogenic acid, which was found in all analyzed samples, typically at the highest levels [82], consistent with previous research.

Although only a limited number of flavonoid and phenolic compounds were identified in this study, the selection was intentional and based on their pharmacological relevance. Compounds such as gallic acid, rutin, chlorogenic acid, ferulic acid, and p-coumaric acid were targeted due to their well-documented biological activities in related plant species. For example, gallic acid and rutin from *Daphne mucronata* have demonstrated antioxidant, antibacterial, hepatoprotective, and nephroprotective properties [1,2]; *Ferula communis* extracts rich in rutin and gallic acid have shown cytotoxic, estrogenic, and anti-inflammatory effects [5,6]; and phenolic acids such as ferulic acid, p-coumaric acid, and vanillic acid in *Heracleum persicum* are associated with antidiabetic and analgesic activities [8,9,10]. Similarly, Tragopogon species have been reported to contain chlorogenic acid and rutin, which contribute to their antioxidant, antimicrobial, and anticancer potential [12,14,15].

The presence of these bioactive compounds may partially explain the traditional therapeutic use of plants in the region. Indeed, the flavonoid and phenolics analysed in this study are not only analytically detectable by HPLC-DAD, but also represent the key flavonoid and phenolic compounds previously associated with therapeutic effects in these plants. Therefore, priority was given to these compounds to establish a meaningful link between traditional use and phytochemical content. Although more advanced analytical techniques such as LC–MS/MS could provide more detailed metabolomic profiles, HPLC-DAD remains a widely used and accessible method for phenolic profiling, particularly when the goal is targeted quantification of known bioactive compounds [2,9,12]. In this study, it allowed for precise detection and steady chromatographic response for target flavonoid and phenolic acids, and was selected based on both scientific suitability and laboratory feasibility.

While this study provides valuable insight into the elemental composition and phenolic content of the selected plants, it does not encompass a full phytochemical characterization. Our analytical focus was guided by the public health relevance of heavy metals and selected antioxidant-related phenolics traditionally associated with these species. Naturally, many other secondary metabolites—such as flavonoids, furanocoumarins, and others—remain to be explored. This should be considered a limitation of the current scope, and future studies may expand on these findings using broader metabolite profiling techniques. More comprehensive phytochemical analyses using advanced techniques such as LC–MS/MS and NMR in the future are likely to reveal additional components. Nevertheless, the findings obtained in this study are intended to provide a scientific basis for future research that will address the metabolomic profiles, pharmacological effects, and safety assessments of the relevant plants in greater detail.

The four medicinal plants (H1–H4) were tested for their antioxidant levels using the DPPH, ABTS, and CUPRAC assays. The results are shown in Table 8. All of the tests showed that the plant extracts were statistically different (*p* < 0.05). *Tragopogon coloratus* (H4) had the most antioxidant activity in all tests, with 88.1% for DPPH, 85.6% for ABTS, and 56.8 µmol TE/g for CUPRAC. This shows that *Tragopogon coloratus* is the most powerful antioxidant source among the studied species, owing to its probable strong radical-removal and electron-transfer capacity. The high antioxidant power of H4 probably comes from its very high amounts of p-coumaric acid (3.40 µg/g) and chlorogenic acid (7.80 µg/g), both of which are known to be powerful antioxidants [83]. Although none of the extracts exceeded the synthetic antioxidant BHT (92.4%), *Tragopoon coloratus* reached nearly 95% of its radical scavenging capacity, indicating probable potential for natural antioxidant use. The dominance of hydroxycinnamic acids such as p-coumaric and chlorogenic acids likely favored electron-transfer (ET) reactions, explaining the strong CUPRAC response [83]. *Heracleum persicum* (H3) ranked second in antioxidant activity (DPPH: 82.4%; ABTS: 79.2%; CUPRAC: 49.7 µmol TE/g). This plant also contained high levels of gallic acid (4.10 µg/g) and rutin (0.83 µg/g), both probably contributing to its antioxidant strength [84]. Rutin, a flavonoid glycoside, can neutralize free radicals and chelate metal ions, in alignment with the observed CUPRAC findings. The combined presence of chlorogenic, p-coumaric, and rutin suggests probable synergistic effects, since these compounds can reinforce each other’s radical-scavenging mechanisms through hydrogen atom transfer (HAT) and single-electron transfer (SET) pathways [84]. *Ferula communis* (H2) also performed well as an antioxidant, particularly in CUPRAC (46.1 µmol TE/g). This is probably due to its high ferulic acid (3.40 µg/g) and p-coumaric acid (5.90 µg/g) contents. Structural studies [84] have shown that hydroxycinnamic acids stabilize radicals through resonance and electron delocalization, enhancing their redox efficiency. *Daphne mucronata* (H1), on the other hand, had the lowest antioxidant activity (DPPH: 66.3%; ABTS: 61.8%; CUPRAC: 33.9 µmol TE/g). Although this species exhibited the highest gallic acid concentration (5.20 µg/g), its radical scavenging potential may have been limited by low chlorogenic (1.10 µg/g) and ferulic acid (0.80 µg/g) levels. In complex plant matrices, probable interactions among phenolic compounds can significantly alter overall antioxidant outcomes; a lack of multiple active phenolics may reduce the total scavenging performance [85]. These findings indicate that the antioxidant capacity of these species is strongly dependent on their specific flavonoid and phenolic compositions. In particular, chlorogenic acid, rutin, and p-coumaric acid appear to be the probable dominant contributors that improve all three antioxidant assays. A positive relationship is typically observed between these hydroxycinnamic acids and radical scavenging parameters, confirming their probable role in both hydrogen-donating and electron-transfer systems. Notably, previous research from the same study found that *Daphne mucronata* accumulated significant quantities of heavy metals, particularly arsenic. When plants are exposed to arsenic, it can induce oxidative stress by generating reactive oxygen species (ROS), which interfere with the biosynthesis of phenolic compounds and related enzymes. Such probable metal-induced oxidative stress can impair antioxidant defense mechanisms, explaining the comparatively weak antioxidant performance of *Daphne mucronata* despite its high gallic acid content [86]. Overall, these results emphasize that phenolic acid diversity—not just total content—probably determines the antioxidant potential of medicinal plants. The observed differences are consistent with redox mechanisms and structure–activity relationships documented for hydroxycinnamic and flavonoid compounds, supporting their use as probable natural antioxidants in nutraceutical and pharmaceutical formulations.

## 4. Conclusions

This study provides an integrated phytochemical, elemental, and toxicological evaluation of four ethnomedicinal plants—*Daphne mucronata*, *Ferula communis*, *Heracleum persicum*, and *Tragopogon coloratus*—traditionally used in the mountainous Hakkari region of southeastern Türkiye. Multiple analytical approaches were employed, including spectrometric methods (AAS and ICP-OES), spectrophotometric assays (DPPH, ABTS, and CUPRAC), chromatographic analysis (HPLC), and microstructural characterization (SEM–EDX). The antioxidant activities were determined using the DPPH, ABTS, and CUPRAC assays, while health-risk assessments for metal exposure were calculated based on Estimated Daily Consumption (EDC), Target Hazard Quotient (THQ), Hazard Index (HI), and Carcinogenic Risk (CR) models. The results showed that *Ferula communis* had the highest phenolic concentration and antioxidant capacity, whereas *Daphne mucronata* accumulated elevated levels of lead, zinc, and copper exceeding WHO/FAO safety limits, which contributed to a higher carcinogenic risk. *Heraclum persicum* and *Tragopogon coloratus* displayed broad phenolic diversity with relatively low metal concentrations, suggesting strong antioxidant potential combined with lower toxicological concern.

The main strength of this work lies in its integrated, multi-method approach that links spectroscopic, chromatographic, and morphological analyses with quantitative health-risk assessment—a combination not previously applied to these species. Nevertheless, the study is limited to leaf tissues and to the flavonoid and phenolic compounds identified by HPLC, offering only a partial view of the plants’ metabolic profiles. Although several phenolic acids with documented therapeutic value were identified, a broader phytochemical investigation would help reveal their full chemical diversity.

Future studies should also explore other plant organs (roots, stems, seeds, and leaves) and investigate additional secondary metabolites using advanced instrumentation such as LC-MS/MS (Liquid Chromatography–Tandem Mass Spectrometry), GC-MS (Gas Chromatography–Mass Spectrometry), and UHPLC-QTOF-MS (Ultra-High-Performance Liquid Chromatography–Quadrupole Time-of-Flight Mass Spectrometry) to provide a more detailed understanding of the bioactive components. Since these species grow naturally at about 2100 m altitude, under high UV radiation and semi-arid soil conditions, they are expected to produce unique secondary metabolites with potential pharmaceutical importance. Isolating and purifying these compounds could pave the way for their use as promising raw materials in drug development. Based on the metal-accumulation patterns observed, certain species—particularly *Daphne mucronata*—were found to absorb higher amounts of toxic elements such as Pb, Zn, and Cu, which may pose health risks if collected from polluted soils. Therefore, regular monitoring of soil and water quality, careful selection of low-contaminant cultivation sites, and controlled harvesting practices are strongly recommended to minimize metal uptake.

Given their pharmacological potential, in vitro tissue culture methods may also serve as a sustainable approach for cultivating these plants under clean, controlled laboratory conditions. Such biotechnological cultivation could help preserve their phytochemical richness while preventing heavy-metal exposure and ensuring the long-term protection of these culturally valuable medicinal species.

Finally, this study emphasizes the two-fold nature of medicinal plants used in Southeast Türkiye (Hakkari): their outstanding therapeutic usefulness and possible toxicological hazards. Recognizing both aspects is essential for transforming long-standing ethnobotanical traditions into evidence-based, safe, and environmentally sustainable health practices.

## Figures and Tables

**Table 1:** Parameters for method validation and analytical conditions of ICP-OES for heavy metals.

Element	Wavelength (nm)	Plasma View	LOD (mg/kg)	LOQ (mg/kg)	RSD (%)	R^2^	Recovery (%)
Al	396.152	Axial	0.300	1.000	1.40	0.9996	91.0
As	193.696	Axial	0.016	0.052	1.35	0.9998	95.0
Cd	228.802	Axial	0.013	0.042	1.07	0.9999	96.8
Co	238.892	Axial	0.020	0.065	1.40	0.9997	91.5
Cr	267.210	Axial	0.022	0.072	1.48	0.9997	93.0
Cu	324.754	Axial	0.050	0.160	1.00	0.9996	92.8
Fe	259.940	Axial	0.390	1.270	1.25	0.9997	90.2
Ni	231.604	Axial	0.028	0.095	1.22	0.9997	93.5
Pb	220.353	Axial	0.018	0.060	0.95	0.9999	94.0
Zn	213.326	Axial	0.180	0.590	1.55	0.9998	95.7

LOD: Limit of detection, LOQ: limit of quantification, CRM: certified reference material, RSD: relative standard deviation.

**Table 2:** Parameters for method validation and analytical conditions of AAS for heavy metals.

Element	Wavelength (nm)	Flame Type	LOD (mg/kg)	LOQ (mg/kg)	RSD (%)	R^2^	Recovery (%)
Ca	422.673	Air/Acetylene	0.52	1.55	1.20	0.9997	91.5
K	766.491	Air/Acetylene	0.21	0.65	1.18	0.9996	92.0
Mg	285.213	Air/Acetylene	0.22	0.66	1.10	0.9996	89.5
Na	589.592	Air/Acetylene	0.11	0.34	1.02	0.9995	92.3

LOD: Limit of Detection, LOQ: Limit of Quantification, CRM: Certified Reference Material, RSD: Relative Standard Deviation.

**Table 5:** Estimated daily consumption (EDC), Target hazard level (THL) and hazard index (HI) for As, Cd, Cr and Pb in infusions of four medicinal plants.

Plant (Code)	Metal	EDC (mg·kg^−1^·Day^−1^)	THL (=EDC/RfD)	HI (ΣTHL)
H1—*Daphne mucronata*	As	1.31 × 10^−4^	4.36 × 10^−1^	
	Cd	2.66 × 10^−6^	2.66 × 10^−3^	
	Cr	2.01 × 10^−4^	6.69 × 10^−2^	
	Pb	4.85 × 10^−4^	1.38 × 10^−1^	0.644
H2—*Ferula communis*	As	3.69 × 10^−5^	6.44 × 10^−1^	
	Cd	8.85 × 10^−7^	1.23 × 10^−1^	
	Cr	4.37 × 10^−5^	8.85 × 10^−4^	
	Pb	3.98 × 10^−5^	1.46 × 10^−2^	0.15
H3—*Heracleum persicum*	As	3.02 × 10^−5^	1.14 × 10^−2^	
	Cd	6.64 × 10^−7^	1.50 × 10^−1^	
	Cr	3.17 × 10^−5^	1.01 × 10^−1^	
	Pb	4.98 × 10^−5^	6.64 × 10^−4^	0.126
H4—*Tragopogon coloratus*	As	3.52 × 10^−5^	1.06 × 10^−2^	
	Cd	7.75 × 10^−7^	1.42 × 10^−2^	
	Cr	3.59 × 10^−5^	1.26 × 10^−1^	
	Pb	4.32 × 10^−−5^	4.36 × 10^−1^	0.142

EDC: Estimated Daily Consumption (mg kg^−1^ day^−1^), THL: Target Hazard Level, HI: Hazard Index, the sum of THL values for each plant. $HI=\sum {THL}_{a}$.

**Table 6:** Estimated lifetime carcinogenic risk of cadmium, chromium and arsenic in adults (68 kg) resulting from infusions of four selected medicinal plants (H1–H4).

Plant	CR—As	CR—Cr	CR—Cd	Total CR
H1—*Daphne*	4.18 × 10^−3^	5.42 × 10^−5^	1.62 × 10^−5^	4.25 × 10^−3^
H2—*Ferula*	1.18 × 10^−3^	1.18 × 10^−5^	5.40 × 10^−6^	1.20 × 10^−3^
H3—*Heracleum*	9.66 × 10^−4^	8.56 × 10^−6^	4.05 × 10^−6^	9.78 × 10^−4^
H4—*Tragopogon*	1.13 × 10^−3^	9.70 × 10^−6^	4.72 × 10^−6^	1.14 × 10^−3^

CR-Lifetime cancer risk.

**Table 7:** Phenolic acid and flavonoid composition of four medicinal plants (H1–H4) determined by HPLC-DAD.

Compounds	H1-*Daphne mucronata*	H2-*Ferula communis*	H3-*Heracleum persicum*	H4-*Tragopogon coloratus*	*p*-Value
Protocatechuic acid	0.35 ± 0.02 ^b^	0.21 ± 0.01 ^c^	0.58 ± 0.03 ^a^	0.12 ± 0.01 ^d^	0.0032
Vanillic acid	0.31 ± 0.02 ^b^	0.18 ± 0.01 ^c^	0.46 ± 0.02 ^a^	0.10 ± 0.01 ^d^	0.0021
Rutin	0.62 ± 0.04 ^b^	0.41 ± 0.03 ^c^	0.83 ± 0.05 ^a^	0.07 ± 0.01 ^d^	0.0004
Gallic acid	5.20 ± 0.30 ^a^	1.80 ± 0.09^d^	4.10 ± 0.20 ^b^	2.50 ± 0.12 ^c^	0.0002
Chlorogenic acid	1.10 ± 0.06^d^	3.60 ± 0.18 ^b^	2.90 ± 0.15 ^c^	7.80 ± 0.40 ^a^	0.0003
p-Coumaric acid	1.70 ± 0.08 ^c^	5.90 ± 0.30 ^a^	5.20 ± 0.26 ^a^	3.40 ± 0.17 ^b^	0.0043
Ferulic acid	0.80 ± 0.05 ^c^	3.40 ± 0.17 ^a^	3.60 ± 0.18 ^a^	1.90 ± 0.09 ^b^	0.0062
o-Coumaric acid	0.55 ± 0.03 ^c^	2.10 ± 0.11 ^a^	1.80 ± 0.09 ^b^	0.95 ± 0.05 ^d^	0.0058

Values are expressed as mean ± SD (n = 3). Different superscript letters within a row indicate significant differences among species (*p* < 0.05). Values are expressed as milligrams per 100 g dry weight of extracts (mg/100 g DW).

**Table 8:** Antioxidant capacities of four medicinal plants.

Sample (Species)	DPPH (% Inhibition)	ABTS (% Inhibition)	CUPRAC (µmol TE/g)
H4-*Tragopogon coloratus*	88.1 ± 1.1 ^a^	85.6 ± 1.2 ^a^	56.8 ± 1.9 ^a^
H3-*Heracleum persicum*	82.4 ± 1.3 ^b^	79.2 ± 1.4 ^b^	49.7 ± 1.7 ^b^
H2-*Ferula communis*	79.6 ± 1.5 ^b^	76.4 ± 1.3 ^b^	46.1 ± 1.6 ^bc^
H1-*Daphne mucronata*	66.3 ± 1.6 ^c^	61.8 ± 1.7 ^c^	33.9 ± 1.8 ^c^
BHT (std., DPPH/ABTS)	92.4 ± 0.8	92.4 ± 0.8	—

BHT was utilized as a standard antioxidant in DPPH and ABTS experiments. CUPRAC and α-Tocopherol techniques referenced Trolox. Statistical letters (a–c) indicate significant sample differences (*p* < 0.05). Values with the same letter are similar. TE: Trolox equivalent.

## Data Availability

Data are contained within the article.
